# Meiotic Chromosome Synapsis-Promoting Proteins Antagonize the Anti-Crossover Activity of Sgs1

**DOI:** 10.1371/journal.pgen.0020155

**Published:** 2006-09-22

**Authors:** Lea Jessop, Beth Rockmill, G. Shirleen Roeder, Michael Lichten

**Affiliations:** 1 Center for Cancer Research, National Cancer Institute, Bethesda, Maryland, United States of America; 2 Department of Molecular, Cellular, and Developmental Biology, Yale University, New Haven, Connecticut, United States of America; 3 Department of Genetics, Yale University, New Haven, Connecticut, United States of America; 4 Howard Hughes Medical Institute, Yale University, New Haven, Connecticut, United States of America; Brandeis University, United States of America

## Abstract

Sgs1, the budding yeast homolog of the mammalian BLM helicase, has been implicated in preventing excess recombination during both vegetative growth and meiosis. Most meiotic crossover (CO) recombination requires full function of a set of yeast proteins (Zip1, Zip2, Zip3, Zip4/Spo22, Mer3, Msh4, and Msh5, termed the SIC or ZMM proteins) that are also required for homologous chromosome synapsis. We report here genetic and molecular assays showing that *sgs1* single mutants display relatively modest increases in CO recombination (less than 1.6-fold relative to wild-type). In contrast, a much greater CO increase is seen when an *sgs1* mutation is introduced into the CO- and synapsis-deficient *zip1, zip2, zip3, mer3,* or *msh4* mutants (2- to 8-fold increase). Furthermore, close juxtaposition of the axes of homologous chromosomes is restored. CO restoration in the mutants is not accompanied by significant changes in noncrossover (NCO) recombinant frequencies. These findings show that Sgs1 has potent meiotic anti-CO activity, which is normally antagonized by SIC/ZMM proteins. Our data reinforce previous proposals for an early separation of meiotic processes that form CO and NCO recombinants.

## Synopsis

Most eukaryotic cells are diploid (two copies of each chromosome per cell), but gametes (in animals, sperm and eggs) are haploid (one chromosome copy). Gametes are produced from diploid cells during meiosis. The two copies of each chromosome are brought together in end-to-end alignment (synapsis), and then are connected by crossover recombination, which involves the joining of DNA from one chromosome copy to DNA of the other. Crossovers are critical for chromosome separation in the diploid-to-haploid transition, and also promote genetic diversity by shuffling parental genotypes.

In contrast, during mitotic cell growth, crossovers create genome rearrangements and loss of heterozygosity, which are associated with cancer and other diseases. A DNA-unwinding enzyme, called BLM in mammals and Sgs1 in budding yeast, prevents mitotic crossover recombination by taking apart intermediates that would otherwise give rise to crossovers.

This paper shows that yeast proteins that promote meiotic chromosome synapsis also protect recombination intermediates from Sgs1. If any of these proteins are absent, Sgs1 prevents both crossover formation and synapsis. These findings show how modulating the activity of a single critical enzyme can either prevent or promote crossover recombination, which threatens genome stability in mitosis but is essential for genome transmission in meiosis.

## Introduction

DNA double-strand breaks (DSBs) pose a significant risk to cells. Failure to repair DSBs can result in death, while imprecise repair can form translocations, deletions, and other chromosome rearrangements. DSBs are repaired by two distinct mechanisms: end-joining, in which the ends of breaks are ligated, often imprecisely, and homologous recombination, in which breaks are repaired using homologous sequences as a template to form recombinants that are either crossover (CO) or noncrossover (NCO) with regard to flanking parental sequences. Although repair by homologous recombination is generally considered nonmutagenic (but see [1]), the CO outcome has the potential for deleterious genome rearrangement, loss of heterozygosity, or both. Perhaps as a consequence, the rare interhomolog recombination events that do occur during the mitotic cell cycle are infrequently accompanied by crossing over [2].

In contrast, COs are frequent in meiosis, with at least one per homolog pair [3]. COs are an integral part of the interhomolog connections that are necessary for homolog alignment and spindle assembly at metaphase I [4,5]. As a consequence, mutants with either general meiotic recombination defects or specific defects in meiotic COs undergo frequent homolog mis-segregation and gamete death. Even a single pair of chromosomes that fails to cross over is at increased risk of nondisjunction at meiosis I [6–8]. In most organisms where these events have been examined, the total number of interhomolog recombination events is considerably greater than the number of COs [9], and both COs and NCOs are needed to facilitate meiotic homolog pairing [10,11].

The molecular mechanism of meiotic recombination and the factors that determine whether events will produce NCO or CO products have been studied most extensively in the budding yeast Saccharomyces cerevisiae. Studies in this organism show that meiotic recombination is initiated by DSBs, formed by the meiosis-specific endonuclease Spo11 [12]. Breaks are subsequently resected to generate single-stranded DNA tails with free 3′ ends [13]. Most COs are produced via formation of a semi-stable single end invasion intermediate in which one DSB end interacts with the homolog [14], followed by capture of the second DSB end to form a double Holliday junction (dHJ) intermediate [14–17]. By contrast, most NCOs form via processes that do not appear to involve stable dHJ intermediates [16,17] and a synthesis-dependent strand-annealing mechanism has been suggested [2,16,18].

Evidence for mechanistic separation of CO and NCO recombination comes from molecular studies of two classes of mutants that block CO formation without reducing NCOs. Cells lacking the Ndt80 transcription factor or the Cdc5 kinase produce NCO recombinant DNA molecules at normal levels, but lack COs and accumulate dHJ intermediates [16,19]. Mutants in any of several budding yeast genes (*ZIP1, ZIP2, ZIP3, ZIP4/SPO22, MSH4, MSH5,* or *MER3,* referred to here collectively as *ZMM* genes) also show CO loss without apparent NCO defects (reviewed in [9]). In strains of the SK1 genetic background that are sporulated at 33 °C, *zmm* mutants display severe defects in single-end invasion and dHJ-intermediate formation as well as CO defects, and this CO loss is not accompanied by an increase in NCO DNA molecules [17]. This finding is consistent with an earlier suggestion that the CO/NCO decision is made at an early step, possibly at or soon after DSB formation [20]. If the decision were made later, blocking CO formation might allow CO-designated DSBs to be repaired as NCOs, which would result in increased NCO production. These two classes of CO-defective mutants also differ in their effect on homolog synapsis, in that *ndt80* and *cdc5* mutants show normal synapsis [19,21], while *zmm* mutants display synapsis defects ([17,22], and references within).

Of the ZMM proteins, Msh4, Msh5, and Mer3 have known biochemical activities that could stabilize early recombination structures and promote the formation of dHJ intermediates [23,24]. The other ZMM proteins appear to participate less directly. Zip1 is a major component of the synaptonemal complex (SC) that forms between homolog axes during prophase of meiosis I [7]. It has been suggested that Zip2 and Zip3 are part of a meiosis-specific ubiquitin- or SUMO-conjugating complex [25–27]. In *zip1*Δ mutants, homolog axes are no longer tightly paired, but instead associate at a few sites per chromosome that are marked by foci of Zip2 and Zip3 [7,22,28,29]. Accumulating data suggest that, in wild-type budding yeast, these Zip2/Zip3 foci, which also contain Msh4 and Msh5 [30,31], mark sites both of CO recombination and of Zip1 polymerization initiation [22,28,29,32,33]. These foci, whose protein contents are termed the synapsis initiation complex (SIC), may correspond to the late recombination nodules that mark CO sites in higher eukaryotes [22,34].

Sgs1, a budding yeast RecQ-type helicase, has been implicated in regulating the CO/NCO decision and in maintaining genome stability. The absence of Sgs1 causes increased mitotic recombination [35], especially when mismatches are present in the recombining partners [36,37]. Mutants lacking Sgs1 also show increased chromosomal rearrangement [38] and reduced sporulation efficiency and spore viability [35]. Similarly, vertebrate cells lacking BLM, an Sgs1 homolog, show elevated rates of sister-chromatid exchange and chromosome rearrangements [39–41]. These mutant phenotypes are consistent with the Sgs1/BLM helicase having a direct anti-CO activity, although many of these observations can also be explained by suggesting that Sgs1/BLM prevents the formation of lesions that provoke recombination or rearrangement.

Consistent with a role for these helicases in directing events away from COs and towards NCOs, human BLM and TOP3α together can dissolve synthetic dHJ substrates in vitro to produce NCOs [42]. While limited solubility has prevented a similar study of Sgs1 [43], two observations support the suggestion that it has anti-CO activity. First, two separate studies, one of spontaneous mitotic recombination and the other of the mitotic repair of a DSB formed by the HO endonuclease, both found about a 2-fold increase in CO recombinants in *sgs1* mutants relative to wild-type, although the vast majority of repair products in both cases were NCOs [44,45]. Second, Rockmill et al. found that, in cells of the BR strain background, the frequency of meiotic COs among tetrads and the number of Zip3–green fluorescent protein (GFP) foci per pachytene nucleus were both about 1.4-fold greater in *sgs1* mutants than in wild-type [32]. Rockmill et al. also showed that *sgs1* mutation accelerates homolog synapsis in otherwise wild-type cells, and restores close, end-to-end association between homolog axes in *zip1* mutants. They referred to the axial association (AA) seen in *zip1 sgs1* as pseudosynapsis, to distinguish it from true synapsis, where end-to-end SC is present.

**Figure 1:**
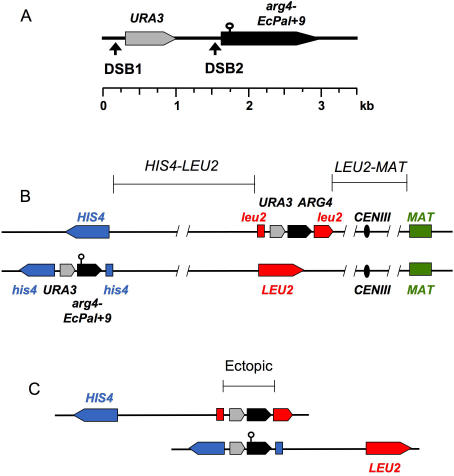
Recombination Interval Used for Molecular Analyses (A) The 3.5-kilobase ectopic recombination interval contains coding sequences for *URA3* (gray) and *ARG4* (black). DSBs form in the promoter regions of *URA3* (DSB1) and *ARG4* (DSB2). The lollipop in *arg4* represents a palindromic sequence inserted at +9 of the open reading frame; this mutation is used to score gene conversion [16]. (B) The ectopic recombination interval is inserted at *HIS4* (blue) on one copy of Chromosome III and at *LEU2* (red) on the homolog. *HIS4* and *LEU2* are 16.7 kilobases apart. In *rad50S* strains, where DSBs persist, 5% of chromosomes have a DSB in *his4::URA3-ARG4* and 0.7% have a DSB in *leu2::URA3-ARG4* [16]. The centromere (black circle) and *MAT* locus (green) are also indicated. Allelic COs can be scored in the *HIS4-LEU2* and *LEU2-MAT* intervals. (C) Ectopic COs can occur between *his4::URA3-ARG4* and *leu2::URA3-ARG4*.

In order to learn more about the role of Sgs1 in meiotic recombination, we examined the effect of *sgs1* mutants on meiotic recombination, using both tetrad analysis and an assay that directly scores recombination at the DNA level (Figure 1). Our findings indicate that, in wild-type cells, Sgs1 activity has a limited role in CO formation and does not play a unique role in NCO formation. However, in *zmm* mutants, where CO formation is markedly reduced and synapsis is impaired, *sgs1* mutants restore COs, in some cases to nearly wild-type levels, and also restore tight homolog AA. These data demonstrate that Sgs1 has anti-CO activity, and suggest that an important role for the SIC/ZMM proteins is to protect nascent CO-designated recombination intermediates from dissolution by Sgs1.

## Results

**Figure 2:**
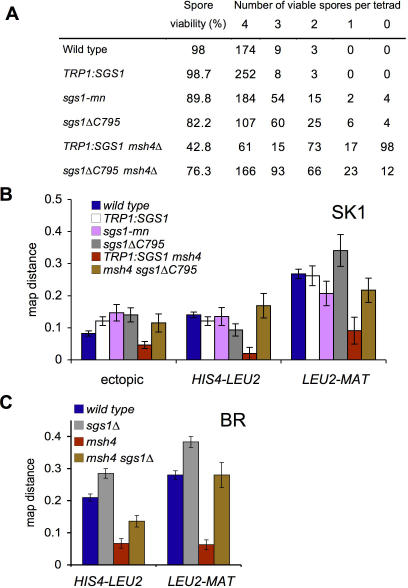
Loss of Full Sgs1 Activity Restores Spore Viability and Crossing Over to *zmm* Mutants (A) Overall spore viability and patterns of spore lethality in tetrads from SK1 strains. (B) Map distance (cM; error bars denote standard error of map distance) in three intervals on Chromosome III in SK1 (see Figure 1B and 1C for details). Values for wild-type are from [16]. (C) Map distance in two allelic intervals on Chromosome III in BR strains. Values for wild-type and *sgs1Δ* are from [32].

Previous studies examined *sgs1*Δ and *sgs1ΔC795* mutants in the BR strain background and found a modest (0%–60%) increase in allelic crossing over, but did not directly evaluate the effect on NCO recombinants ([32]; B. Rockmill, K. Voelkel-Meiman, and G. S. Roeder, unpublished data). In the SK1 background, homozygous *sgs1Δ* diploids display high chromosome instability, and mating-type heterozygosity cannot be maintained at levels that ensure sporulation in liquid culture. To extend evaluation of the meiotic role of Sgs1 to SK1 strains, where recombination can be readily scored at the DNA level, we used two *sgs1* mutant alleles, *sgs1ΔC795* and *sgs1-mn*. Neither allele displays the same extent of chromosome instability as *sgs1Δ*, and both support efficient premeiotic growth and sporulation. The *sgs1ΔC795* allele expresses only the first 652 amino acids of the protein and is lacking both the helicase domain [46] and a region called the HRDC domain, which in BLM interacts with Holliday junctions [42]. A DNA fragment containing *SGS1* or *sgs1ΔC795* coding sequences along with 600 nucleotides of upstream sequences was integrated at *TRP1* in strains where the endogenous *SGS1* gene was deleted (see Materials and Methods). We will refer to strains with *sgs1ΔC795* at *TRP1* as *sgs1ΔC795,* and to isogenic control strains with *SGS1* at *TRP1* as *TRP1:SGS1.* “Wild-type” will be reserved for strains with *SGS1* at its normal locus. In the *sgs1-mn* allele, *SGS1* is transcribed from a *CLB2* promoter, which is expressed during the mitotic cell cycle but not during meiosis. *TRP1:SGS1* and wild-type strains show similar spore viability (≥98%; see also Figure 2A) and resistance to methyl methane sulfonate (unpublished data). Both *sgs1ΔC795* and *sgs1-mn* show reduced spore viability (Figure 2A), with spore inviability patterns typical of random spore death. A substantial fraction of this spore death is likely to be due to premature separation of sister chromatids, associated with recombination near centromeres (B. Rockmill, K. Voelkel-Meiman, and G. S. Roeder, unpublished data).

### Sgs1 Has a Limited Effect on CO Recombination in Wild-Type Cells

We examined meiotic recombination in SK1 strains carrying a 3.5-kilobase *URA3-ARG4* recombination interval inserted at *his4* on one copy of Chromosome III and at *leu2* on the homolog (Figure 1). COs in three intervals can be scored in these strains: ectopic COs between the *his4::URA3-ARG4* and *leu2::URA3-ARG4* inserts, allelic COs in the *HIS4-LEU2* interval, and allelic COs in the *LEU2-MAT* interval. *TRP1:SGS1* strains displayed a 1.4-fold greater frequency of ectopic COs when compared with wild-type (*p* < 0.001, *G*-test), but CO frequencies in the two allelic intervals were similar to those seen in wild-type (Figure 1B). Both *sgs1* mutants produced ectopic COs at frequencies similar to those seen in *TRP1:SGS1* and about 1.7-fold greater than in wild-type. Allelic CO frequencies in *sgs1* mutants were not consistently greater or less than those seen in wild-type or *TRP1:SGS1,* and in all cases were within 30% of wild-type or *TRP1:SGS1* values (Figure 2B). In the BR background, *sgs1Δ*-null mutants and *sgs1ΔC795* display consistent increases of 30%–40% in allelic crossing over in the *HIS4-LEU2* and *LEU2-MAT* intervals [32].

The Zip3 protein forms foci on pachytene chromosomes, and it has been suggested that, in wild-type cells, these foci mark sites of COs [22]. In the BR strain background, *sgs1Δ*-null mutants and *sgs1ΔC795* mutants display a 1.5- and 1.3-fold increase, respectively, in the number of Zip3 foci, detected using a Zip3-GFP fusion protein ([32]; B. Rockmill, K. Voelkel-Meiman, and G. S. Roeder, unpublished data). Loss of functional Sgs1 protein has a similar impact on Zip3-focus formation in the SK1 background. Comparable numbers of Zip3-GFP foci were detected in wild-type and *TRP1:SGS1* nuclei (wild-type 63 ± 8 foci/nucleus, 34 nuclei scored; *TRP1:SGS1* 66 ± 7 foci/nucleus, 40 nuclei scored), while about 25% more foci were detected in *sgs1ΔC795* nuclei (82 ± 9 foci/nucleus, 39 nuclei scored; *p* < 0.001, *t*-test).

**Figure 3:**
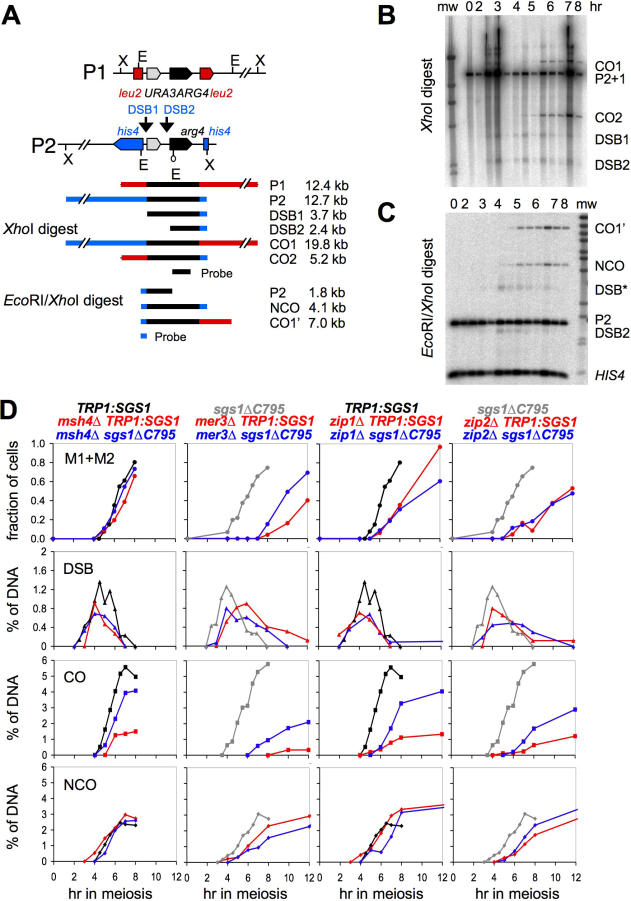
Sgs1 Prevents COs in *zmm* Mutants (A) Schematic representation of the ectopic *URA3-ARG4* interval. Symbols are as in Figure 1. EcoRI (E) and XhoI (X) restrictions sites are indicated. To detect COs and DSBs, DNA is digested with XhoI and probed with *ARG4* sequences (ArgD; [16]). To detect NCOs and a CO product (CO1′), DNA is digested with EcoRI and XhoI and probed with *HIS4* sequences (HisU; [16]). (B) Southern blot of DNA isolated from a meiotic culture of MJL3035 (*TRP1:SGS1*) at the indicated time after initiation of sporulation, digested, and probed to detect COs and DSBs. mw, HinDIII digest of phage λ DNA. DSBs occur in *URA3-ARG4* inserted at both *his4* and *leu2,* but are ~7-fold stronger in the *his4* insert than at *leu2* [16]. Palindrome cleavage, by unidentified activities, occurs at the same time as CO formation and results in a band about the size of DSB2 (T. Allers, L. Jessop, and M. Lichten, unpublished data). (C) Southern blot of the same samples, digested and probed to detect NCO and CO1′ recombinants. *HIS4*, *HIS4* locus lacking an insert; DSB*, DSB1 product where resection has passed the EcoRI site in *HIS4*; mw, BstEII digest of phage λ DNA. (D) Molecular analysis of mutants. Color codes: *TRP1:SGS1,* black; *sgs1ΔC795,* grey; *TRP1:SGS1 zmm* single mutants, red; *sgs1ΔC795 zmm* double mutants, blue*.* M1+M2, percent of cells containing at least two nuclei, a sign of passage through meiosis 1. DSB, DSB1 band signal/total lane signal from Southern blots as in (B). CO, CO1′ signal/total lane signal from Southern blots as in (C). NCO, NCO band signal/total insert signal from Southern blots as in (C).

Most tetrad analyses consider only data from four-spore viable tetrads. Because COs promote spore viability, this can overestimate CO frequencies in mutant backgrounds in which spore viability is reduced. To examine every meiotic product regardless of viability, and to determine whether or not Sgs1 function affects NCO formation, we scored recombinants in the ectopic *URA3-ARG4* interval at the molecular level, using DNA from SK1 cultures undergoing synchronous meiosis (Figure 3).

Timing of DSB appearance and disappearance, maximal levels of DSBs, and timing of meiotic divisions did not differ substantially among wild-type, *TRP:SGS1, sgs1ΔC795,* and *sgs1-mn* (Figure 3D and unpublished data). Molecular analysis revealed no statistically significant difference among wild-type, *TRP1:SGS1, sgs1ΔC795,* or *sgs1-mn* strains with regards to the timing of formation or final levels of NCOs or COs in the ectopic recombination interval (Figures 3D and S2), although experiment-to-experiment variation would have obscured CO increases of 30% or less. These results indicate that, in otherwise wild-type SK1 cells, the majority of CO and NCO recombinant molecules form independently of full Sgs1 function.

We performed a similar analysis in strains containing a different recombination interval, *URA3-tel-ARG4,* integrated at *HIS4* and *LEU2* [18]. This interval differs from the *URA3-ARG4* interval described above in that DSBs occur at a single site in the interval and form more frequently (20% versus 5% of chromosomes). Wild-type, *TRP1:SGS1,* and *sgs1ΔC795* strains produced similar levels of NCO and CO recombinant molecules in this interval (Figure S3).

### Sgs1 Inhibits CO Formation in *zmm* Mutants

The finding that Sgs1 has a limited impact on meiotic recombination in wild-type SK1 cells stands in contrast to numerous reports suggesting a prominent anti-CO function during the mitotic cell cycle. Because SIC/ZMM proteins promote meiotic CO formation, we reasoned that they might be masking the anti-CO activity of Sgs1. Therefore, we examined the effect of *sgs1* mutation on meiotic recombination in several *zmm* mutants in SK1 and BR strains. The results of these studies are summarized below.

#### msh4Δ.

SK1 *msh4* mutants show relatively high sporulation frequencies and spore viability [31], allowing tetrad-based measurements of genetic distances. Consistent with previous studies [31], *TRP1:SGS1 msh4Δ* strains showed about 2.5-fold fewer COs compared with *TRP1:SGS1 MSH4,* in all three intervals illustrated in Figure 1, a marked reduction in spore viability, and a disproportionate increase in the number of tetrads with two or no viable spores (Figure 2A and 2B). All three phenotypes were suppressed by *sgs1ΔC795* (Figure 2), with similar spore viability patterns and CO frequencies seen in *sgs1ΔC795 MSH4* and *sgs1ΔC795 msh4*Δ. A similar *msh4Δ* CO defect, and suppression by *sgs1Δ,* was seen in BR strains (Figure 2C).

Molecular assays confirm this CO defect, and its suppression by *sgs1* mutation. COs in *TRP1:SGS1 msh4*Δ were reduced 3.7-fold relative to *TRP1:SGS1* (Figure 3D). A similar reduction was seen in *msh4Δ* (Figure S4). COs were increased nearly to *MSH4* levels in *sgs1ΔC795 msh4*Δ or *sgs1-mn msh4Δ* (2.7-fold greater than *TRP1:SGS1 msh4*Δ or *msh4Δ* alone; Figures 3 and S4). We found no substantial differences in the time of formation or in final levels of NCO recombinants between *msh4Δ* and control strains. The *msh4Δ* mutant also had no defects in DSB formation, DSB repair, or meiotic progression.

#### mer3Δ.

Unlike *msh4Δ, mer3Δ* cells show DSB repair and meiotic progression defects (Figure 3; [47]). DSBs formed normally in both *TRP1:SGS1 mer3Δ* and *sgs1ΔC795 mer3Δ* strains, but some breaks persisted beyond the normal time of repair, with DSBs detectable in TRP1:*SGS1 mer3*Δ cells after 12 h of sporulation. This DSB repair defect was partially suppressed by *sgs1ΔC795,* with all DSBs gone after 10 h of sporulation. Meiotic progression was also defective in *TRP1:SGS1 mer3,* with binucleate cells appearing 3 h later than normal (Figure 3D), and only about 40% of cells completing meiosis I by 12 h. A greater fraction of *sgs1ΔC795 mer3Δ* cells completed at least one division at 12 h, although progression was still delayed.

The CO defect we observed in *TRP1:SGS1 mer3Δ* strains was more severe than the 2- to 3-fold reduction previously reported [47]. COs could not be detected in *TRP1:SGS1 mer3Δ* mutants 8 h after initiation of sporulation, a time when COs had reached a maximum in *TRP1:SGS1 MER3*. Even at 12 h, CO levels were 17-fold less than in control strains. This CO defect was partially suppressed by *sgs1ΔC795,* and COs in *sgs1ΔC795 mer3Δ* were 7-fold greater than in *TRP1:SGS1 mer3* at 12 h. Nevertheless, CO levels reached only about 40% of the maximum level seen in *MER3* controls*.* A modest NCO defect was also seen in *TRP1:SGS1 mer3Δ*. After 8 h of sporulation, NCO levels in both *TRP1:SGS1 mer3Δ* and *sgs1ΔC795 mer3*Δ were less than those seen in *TRP1:SGS1 MER3,* although NCO frequencies reached or exceeded those seen in *TRP1:SGS1 MER3* by 12 h.

#### zip1Δ and zip2Δ.

DSB formation occurred on time, and most DSBs were repaired in *zip1*Δ and *zip2*Δ strains. These strains also showed delayed meiotic progression that was not affected by *sgs1ΔC795* (Figure 3). At 8 h after initiation of sporulation, COs were present in *TRP1:SGS1 zip1*Δ at 20% of the maximum level seen in *TRP1:SGS1 ZIP1*. The *sgs1ΔC795* allele partially suppressed this defect, increasing COs 3-fold. NCO formation was unaffected. *TRP1:SGS1 zip2Δ* mutants displayed a more severe CO defect. At 8 h, COs were present at 11% of the maximum seen in *TRP1:SGS1 ZIP2*. At 12 h, COs levels had increased another 2-fold. COs were increased about 3-fold by *sgs1ΔC795* in *zip2Δ,* as they were in *TRP1:SGS1 zip1Δ*. As was seen in *TRP1:SGS1 mer3*Δ*, TRP1:SGS1 zip2*Δ caused a slight delay and reduction in NCOs that was not suppressed by *sgs1ΔC795*; in both *zip2*Δ *TRP1:SGS1* and *zip2Δ sgs1ΔC795,* NCOs continued to accumulate and at 12 h their level exceeded the maximum seen in *TRP1:SGS1* at the same time (Figure 3).

We also considered whether *sgs1Δ* suppresses the allelic CO defect seen in *zip1Δ* in the BR strain background. Because *zip1Δ* mutants sporulate poorly in BR, map distances were measured by random spore analysis; *sgs1Δ* caused about a 2-fold increase in COs in both intervals (Table S2).

In summary, in the conditions used in these experiments, *zmm* mutants differ in terms of meiotic progression, DSB repair, and CO formation defects, consistent with the diversity of defects previously seen when SK1 *zmm* mutants are sporulated at 23 °C [17]. Despite these differences, in all cases the *zmm* mutant phenotype is at least partially suppressed by loss of Sgs1.

### Sgs1 Inhibits Homolog AA in *mer3Δ* and *zip3Δ* Mutants

Despite the absence of Zip1, the protein that normally occupies the space between synapsed homolog axes [48], close juxtaposition of homolog axes along their lengths is observed in *sgs1 zip1* double mutants in both BR and SK1 strain backgrounds ([32] and unpublished data). To determine whether this pseudosynapsis occurs in other *zmm sgs1* double mutants, we examined chromosome morphology in surface-spread meiotic nuclei, using antibodies against Zip1 and antibodies against Red1, a major component of meiotic chromosome axes. In wild-type yeast, chromosome cores never achieve fully continuous Red1 staining [49]. However, in synapsis-defective *zmm* mutants, Red1 accumulates and localizes continuously along each chromosome axis [29]. Thus, Red1 staining provides a means to visualize chromosome contours in the absence of Zip1 staining.

In SK1 strains, both *TRP1:SGS1* and *sgs1ΔC795* displayed normal chromosome morphology, with axes of homologous chromosomes closely juxtaposed and continuous end-to-end Zip1 staining (unpublished data). However, both strains displayed an increased frequency of polycomplexes (extrachromosomal arrays of SC components) compared with wild-type (28/83 nuclei in *TRP1:SGS1* and 34/74 nuclei in *sgs1ΔC795* versus 4/50 nuclei in wild-type after 5 h of sporulation). This increase in polycomplex formation suggests a modest defect or delay in SC formation.

**Figure 4:**
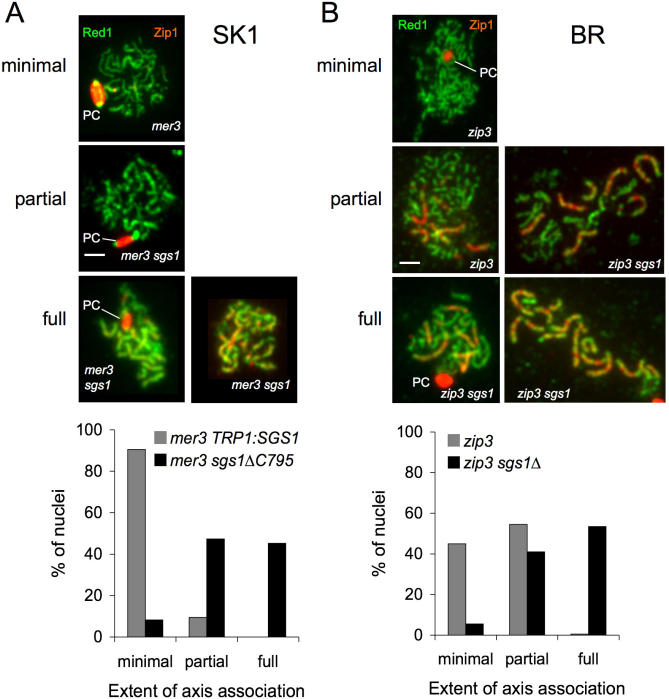
Sgs1 Prevents AA in *mer3Δ* and *zip3Δ* Mutants (A) Analysis of AAs in TRP1:SGS1 *mer3Δ* and *mer3Δ sgs1ΔC795* SK1 strains. Nuclei from cells harvested 5 h after initiation of sporulation were surface spread and probed with anti-Zip (red) and anti-Red1 (green) antisera. Nuclei where chromosomes displayed linear Red1 were examined and classified as displaying minimal, partial, or full pseudosynapsis, as described in Materials and Methods. Nuclei with fully associated chromosomes displayed discontinuous Zip1 staining (right-hand example), Zip1 localization in polycomplexes (PC, left-hand example), or both. White bar: 2 microns. (B) Analysis of AAs in *zip3Δ* and *zip3Δ sgs1Δ* BR strains. Nuclei from cells harvested 18 h after the initiation of sporulation were spread, stained, and analyzed as for *mer3* strains. In most nuclei with full AA, at least some chromosome pairs displayed end-to-end Zip1 staining with the remainder being pseudosynapsed.

*TRP1:SGS1 mer3Δ* diploids displayed a severe synapsis defect, with about 90% of nuclei showing separated homolog axes (as evidenced by Red1 staining) and little Zip1 localization (Figure 4). Homolog AA was partially restored in *mer3Δ sgs1ΔC795* mutants, with more than 90% of cells displaying either some or all chromosomes with axes in close alignment along their lengths (Figure 4A). These fully aligned chromosomes displayed only low levels of discontinuous Zip1 staining, in contrast to the end-to-end continuous staining seen in *sgs1* single mutants and in wild-type ([7,17,32] and unpublished data). Although about half of *mer3Δ sgs1ΔC795* nuclei displayed full pseudosynapsis of homologs, an equal number displayed partial pseudosynapsis, where only some homologs or parts of homologs appeared pseudosynapsed.

We also determined the effect of Sgs1 on synapsis in *zip3Δ* BR strains, which display a relatively mild CO and progression defect [28]. Only a small fraction of *zip3* mutant cells displayed full homolog AA, and this occurred at relatively late times in meiosis. Despite this synapsis defect, about 75% of *zip3Δ* mutant cells progress to form mature asci (unpublished data). Deletion of *SGS1* from *zip3Δ* mutants restored full homolog AA to more than half of nuclei (Figure 4B). Of the chromosome pairs in which axes were closely juxtaposed, most were truly synapsed (i.e., displayed end-to-end Zip1 staining) and a minor fraction were pseudosynapsed (Figure 4B and unpublished data).

## Discussion

In previous studies of BR strains, Rockmill et al. showed that the *sgs1*Δ and *sgs1*Δ*C795* mutations increase crossing over in allelic intervals (1.2- to 1.4-fold) and cause a corresponding increase (1.3- to 1.4-fold) in the number of Zip3 foci, which are thought to be cytological markers of CO sites [32]. In this study, we examined the effect of *sgs1* mutations on meiotic recombination in SK1 strains, using assays that detected NCO and CO DNA molecules produced by ectopic recombination. We did not observe a statistically significant increase in CO molecules in two test intervals, although experiment-to-experiment variation would have obscured an increase of 30% or less. We also did not observe a consistent increase in CO recombination, measured by tetrad analysis in three genetic intervals on Chromosome III. We did observe an increase (by about 25%) in Zip3 foci in *sgs1ΔC795* SK1 strains (relative to *TRP1:SGS1*). If Zip3 foci are accurate markers of CO sites, this finding would be consistent with a modest increase in crossing over on a genome-wide basis. In a separate study, Oh and Hunter have examined the effect of *sgs1ΔC795* on CO recombination in SK1 diploids, using both genetic and molecular assays. Their data indicate that, in the SK1 background, loss of *SGS1* function increases overall CO frequencies by no more than 20% (S. Oh and N. Hunter, personal communication).

We also found that, in SK1 *sgs1* mutants, NCO recombinants are recovered at frequencies similar to those seen in wild-type; similar results have been observed in BR strains [32]. Thus, the bulk of the genetic, DNA-based, and cytological assays suggest that, in wild-type cells, there must be proteins other than Sgs1 that prevent COs, and that promote NCO recombination.

### Sgs1 Mediates the CO Defect in *zmm* Mutants

COs are required for proper chromosome segregation during meiosis, and both meiotic CO distributions and the time of their formation are tightly regulated in most organisms [9,50]. It is therefore likely that the anti-CO activity of the Sgs1/BLM helicase evident in mitotic cells [35–37,39–41] must be modulated during meiosis to allow high CO levels, either by reducing helicase activity or by preventing access to substrates. Our current study identifies SIC/ZMM proteins encoded by the budding yeast *ZIP1, ZIP2, ZIP3, MER3,* and *MSH4* genes, as modulators of the impact of Sgs1. Although mutations in these genes reduce CO formation, the severity of this defect can vary widely. For example, under the sporulation conditions used in our study, this CO defect ranged from about a 4-fold reduction (in *msh4Δ*) to a virtual elimination (in *mer3Δ*). These differences most likely reflect the different biochemical and structural roles played by the different ZMM proteins, either individually or as part of a larger complex. Nevertheless, the CO defect in these mutants was at least partially suppressed by the loss of Sgs1 activity. In molecular assays, CO restoration was not accompanied by a corresponding decrease in NCO recombinants, and COs were not restored to levels seen in wild-type or in the *sgs1ΔC795* single mutant. Genetic assays, in both SK1 and BR backgrounds, also showed substantial restoration of COs to *zmm* mutants by *sgs1* mutation. The *sgs1ΔC795* mutation also has been found, in genetic and molecular assays, to restore crossing over to *msh5* and *mlh3* mutants in the SK1 background (S. Oh and N. Hunter, personal communication). These findings indicate that Sgs1 can act specifically to prevent CO formation during meiosis, but that this activity is primarily manifest in cells lacking intact SIC/ZMM protein function. Below, we briefly consider mechanisms by which Sgs1 might decrease COs in *zmm* mutants.

#### COs restored in *zmm sgs1* double mutants come from NCOs.

Allers and Lichten suggested that CO and NCO recombinants are the products of alternate processing of an early strand invasion intermediate [16]. Helicase-driven destabilization of this intermediate would produce NCOs via a process similar to synthesis-dependent strand annealing; stabilization would allow capture of the second break end, producing a dHJ intermediate that subsequently would be resolved as a CO. This model predicts that CO increases in *zmm sgs1* mutants should be accompanied by equivalent decreases in NCOs. This prediction is not supported. There is no decrease in NCO levels in *msh4 sgs1* and *zip1 sgs1* strains compared with *msh4* and *zip1* single mutants, respectively. The slight decrease in NCOs in *mer3 sgs1* and *zip2 sgs1* strains compared with the *mer3* and *zip2* single mutants, respectively, cannot account completely for the restoration of COs (Figure 3). We therefore consider the alternate processing hypothesis to be unlikely.

#### COs in *sgs1 zmm* double mutants depend on Mus81/Mms4.

It has been suggested that, in *S. cerevisiae,* most meiotic COs are ZMM-dependent, with a minor fraction being produced by a ZMM-independent pathway that requires Mus81/Mms4 endonuclease activity to resolve recombination intermediates as COs [51,52]. One way to account for partial CO restoration in *sgs1 zmm* double mutants would be to suggest that Sgs1 activity blocks this ZMM-independent pathway. If this putative second pathway were completely separate from ZMM-dependent processes, then all *sgs1 zmm* mutants should display a similar increase in COs, which we do not observe. However, our data do not exclude the possibility that, in the absence of Sgs1 activity, resolution of intermediates as COs requires Mus81/Mms4 activity. Experiments to test this possibility are ongoing.

#### ZMM proteins protect pre-CO intermediates from Sgs1.

ZMM proteins colocalize in foci whose number and distribution are similar to those of meiotic COs, and it has been suggested that these foci correspond to the late recombination nodules observed in higher eukaryotes that mark sites of crossing over [22]. One possible function for these large structures would be to promote progression of recombination intermediates that are designated to produce COs [17], perhaps by shielding them from Sgs1 and other helicases, or by modifying Sgs1 so that it no longer has anti-CO activity. If this were the case, then the presence or absence of either ZMM proteins or fully functional Sgs1 should not substantially alter NCO levels. Our data are consistent with this suggestion, and thus provide further support for previous suggestions that NCOs and COs diverge at a very early step in meiotic recombination, and that only limited crosstalk occurs between the two pathways once the separation occurs [17,20].

What happens to the DSBs and recombination intermediates that would have given rise to COs in *zmm* mutants? Some of these mutants display persistent unrepaired DSBs, which could come either from intermediates disassembled in vivo, or from intermediates, arrested at the strand invasion stage, that fall apart during DNA preparation. However, this is not universally true. In *msh4Δ* at 30°C, COs are reduced significantly, yet DSBs do not visibly persist, and cells progress normally through both meiotic divisions. In this case, the DSBs that would have given rise to COs must have been repaired. Because COs are reduced, and NCOs are not increased in this mutant, it is likely that these “missing breaks” are repaired by sister-chromatid recombination, which would be undetected in our assays.

### Sgs1 Inhibits Interhomolog AA

In *zip1* single mutants, the cores of each pair of homologous chromosomes are connected at only a few sites, referred to as AAs [7,32]. The number of AAs is increased in *zip1 sgs1* double mutants to the point where homolog axes appear to be tightly associated along their lengths [32]. This *sgs1* effect appears to be a general phenomenon for *zmm* mutants, as increased association between homolog axes occurs in *mer3Δ sgs1ΔC795* and *zip3 sgs1Δ* double mutants (this study), and in *zip2 sgs1* and *zip4 sgs1* double mutants (B. Rockmill and G. S. Roeder, unpublished data). Zip1 is present in these mutants, and the additional homolog association promoted by *sgs1* mutation is often accompanied by regions of normal synapsis (i.e., Zip1 assembly), although synapsis is frequently incomplete.

A previous study has reported that, in *zip1 sgs1* double mutants, the increase in the number of AAs appears to be much greater than the increase in the number of COs [32]. Our cytological study of *mer3Δ TRP1:SGS1* and *mer3Δ sgs1ΔC795* provides further evidence for a discrepancy between the number of COs and the number of AAs. In particular, *mer3Δ sgs1ΔC795* double mutants show complete pseudosynapsis in about half of cells, but COs are restored to levels that are only 1.4-fold greater than those seen in *zip1Δ TRP1:SGS1* (Figure 3 and unpublished data), where axes associate at only a few points per chromosome. It therefore appears likely that, when full Sgs1 activity is absent, AAs occur at more sites than the ones that give rise to COs.

The molecular nature of the interhomolog interactions at these association sites remains to be determined. However, it is likely that it involves recombination intermediates, since the formation of AAs depends on DSBs [53] and AAs are observed in SK1 strains at a time when most mature CO products have not yet appeared (Figure 3). Because the number of AAs seen in *zip1* single mutants approximates the number of COs seen in wild-type [29], it is unlikely that the additional AAs seen in *zip1Δ sgs1ΔC795* or in *mer3Δ sgs1ΔC795* double mutants reflect interhomolog interactions that will eventually be processed to form COs. Instead, we suggest that they contain recombination intermediates that either are resolved as NCOs, or are disassembled and repaired by sister-chromatid recombination later in meiosis. Further study will be required to determine which of these suggestions is correct.

In summary, the data presented here point to a mutual antagonism between Sgs1 and functions that promote homologous chromosome colocalization and synapsis during meiosis. We suggest that, on one hand, the SIC/ZMM proteins prevent Sgs1 from disassembling nascent CO-designated intermediates; on the other hand, Sgs1 activity may limit stable, long-lived associations between homologous chromosomes to sites that will be used for COs.

## Materials and Methods

### Strains and media.

Strains used for molecular analyses (Table S1A) are all direct derivatives of SK1 [54]. The *URA3-ARG4* recombination interval used has been described previously [16]. Strain construction details are given in Protocol S1. Deletions of *SGS1, MSH4, MER3, ZIP1,* and *ZIP2* were made by replacing coding sequences with a G418-resistance cassette [55]. Strains with *sgs1ΔC795* contain this allele integrated at *TRP1* and the endogenous *SGS1* locus deleted; as controls, strains with *SGS1* at *TRP1* were used. The meiotic null allele of *SGS1 (sgs1-mn)* was made as described [56]. These *sgs1-mn* mutants grow as well as wild-type in the presence of 0.012% MMS, which prevents growth of *sgs1*-null mutants, indicating that *sgs1-mn* retains normal mitotic function. Quantitative Western blots showed that the 3HA-Sgs1 protein expressed from *sgs1-mn* is rapidly degraded during meiosis: 2 h after induction of sporulation, about 10% of the protein remains, and none remains after 4 h (Figure S1).

Zip3 foci were quantified in strains that were heterozygous for an insertion of pSA219 (*ZIP3-GFP-URA3;* [28]). Strains homozygous for this insert displayed greater than normal spore lethality, and were therefore not used.

Genetic and cytological analyses were also done with strains isogenic to BR1919-8B (Table S1B). Wild-type, *sgs1::KAN, zip3::URA3,* and *msh4::ADE2* strains have been described [22,32]. Genetic crosses were used to make double mutants.

### Genetic and molecular analyses.

Yeast media and genetic procedures were as described [18]. Genetic distance determinations (map distance in cM and standard error of map distance) used the calculator at http://www.molbio.uoregon.edu/~fstahl. Only tetrads with four viable spores were considered. *G*-test analysis of tetrad class distributions used to calculate map distances used a Microsoft Excel calculator kindly supplied by E. Hoffmann and R. Borts. Recombination intermediates and recombination products were detected and quantified as described [16,18].

### Cytological analysis.

Nuclear spreads were performed as described [29], with antibody staining and Zip3 focus quantification as described [22,28,32]. In *mer3Δ* and *mer3Δ sgs1ΔC795* strains, Zip1 does not assemble properly into SC ([17] and this paper), so chromosome association was evaluated by examining nuclear spreads in which Red1 staining was continuous. AAs were scored as “minimal” if chromosomes consisted of thin (i.e., single) axes with only a few points of association, as “full” if the majority of chromosome axes were thick (i.e., clearly doubled), and as “partial” if they displayed a morphology intermediate between these two states (see Figure 4). 200 nuclei were scored for each mutant genotype.

## Supporting Information

Figure S1 *sgs1-mn* Is a Meiosis-Specific Null Allele (A) Western blot probed with anti-HA (top panel) to detect 3HA-Sgs1 expressed from the *CLB2* promoter, or anti-Tub2 (bottom panel) to detect Tub2 as a loading control. Protein was extracted from a synchronously sporulating culture of MJL3091 at the indicated times. * indicates cross-reacting protein that is present in all samples.
(B) Graph of relative 3HA-Sgs1 levels, with 0 h sample levels set at 100%. This corresponds to between 1 and 1.5 times the level of Sgs1 seen in 0 h samples from wild-type cells (unpublished data).
(1.0 MB TIF)

Figure S2 Effect of *sgs1ΔC795* on CO Recombination in SK1 Strains CO recombination was measured as described in Figure 3B. Values reflect averages of 7 and 8 h samples from multiple blots of DNA from several independent cultures. Number of determinations were as follows: wild-type, 18 measurements, 5 cultures; *TRP1::SGS1,* 8 measurements, 3 cultures; *sgs1ΔC795,* 6 measurements, 3 cultures; *sgs1-mn,* 5 measurements, two cultures.
(330 KB TIF)

Figure S3 *sgs1ΔC795* Does Not Substantially Alter CO or NCO Recombination in a Second Interval CO and NCO recombination were measured in a *URA3-tel-ARG4* recombination reporter insert [8] at *LEU2* and *HIS4* on parental homologs.
(A) Structure of the insert and detection of recombinants. In this insert, *URA3* and *ARG4* are in opposite orientations, and recombination is initiated at a single DSB site, promoted by a 60 nucleotide insert containing telomere repeats *(tel)*. CO1 and NCO recombinants were detected essentially as described in Figure 3, by digesting and probing as follows: CO1: XhoI digest, probe with *ARG4* sequences (black box); NCO: EcoRI/XhoI digest, probe with *his4′* sequences (blue box).
(B) Average CO and NCO product frequencies from 7 and 8 h samples for wild-type (MJL2984), *TRP1::SGS1* (MJL3033), and *sgsΔC795* (MJL3034) strains. Bars indicate standard deviations for the following number of determinations: wild-type: CO 4, NCO 2; *TRP1::SGS1:* CO 3, NCO 4; *sgsΔC795:* CO 4, NCO 3.
(924 KB TIF)

Figure S4 An *sgs1* Meiotic Null Mutant Restores COs to *msh4Δ* Mutants Cultures of *msh4Δ* (MJL3120, red), *sgs1-mn* (MJL3091, black), and *msh4Δ sgs1-mn* (MJL3124, blue) were sporulated, and samples taken at the indicated times were analyzed for nuclear divisions (MI + MII), DSBs, and CO and NCO recombinants (NCO and CO1′) as in Figure 3C.
(719 KB TIF)

Protocol S1 Supplementary Online Methods (37 KB DOC)

Table S1 Strain Genotypes (77 KB DOC)

Table S2 *sgs1Δ* Restores Crossovers to a *zip1Δ* Mutant in the BR Strain Background (32 KB DOC)

### Accession Numbers

The UniProt (http://www.pir.uniprot.org) accession numbers for the proteins mentioned in this paper are BLM helicase (P54132), Cdc5 (P32562), Mer3/Hfm1 (P51979), Msh4 (P40965), Msh5 (Q12175), Ndt80 (P38830), Red1 (P14291), Sgs1 (P35187), Spo11 (P23179), TOP3 alpha (Q13472), Tub2 (P02557), Zip1 (P31111), Zip2 (P53061), Zip3/Cst9 (Q06032), and Zip4/Spo22 (P40511).

## Abbreviations

AA: axial association
CO: crossover
dHJ: double Holliday junction
DSB: double-strand break
NCO: noncrossover
SC: synaptonemal complex
SIC: synapsis initiation complex
ZMM: Zip Msh Mer proteins
