# Supplementary material for: Meiotic Chromosome Synapsis-Promoting Proteins Antagonize the Anti-Crossover Activity of Sgs1
Source: PLoS Genet. 2006 Sep 22;2(9):e155. doi: 10.1371/journal.pgen.0020155 (PMC1570379; doi:10.1371/journal.pgen.0020155)
Supplement: Supplementary file 1 [file pgen.0020155.sd001.doc]

**Supplementary Online Methods Protocol 1**

**Strain construction**. SK1 strains are listed in Table S1. Deletions of *SGS1*, *MSH4*, *MER3*, *ZIP1* and *ZIP2* were constructed as follows: the desired deletion/replacement allele plus 200-300 nt of flanking genomic sequences on both sides was PCR-amplified (primer sequences supplied upon request), using template DNA from a Yeast Deletion Collection strain (Open Biosystems). SK1 strains were transformed with these fragments as described [1]. Transformants were verified by PCR or on Southern blots.

The *sgs1∆C795* allele was integrated at *TRP1* as follows: a *Not*I fragment from pAG32 [2] was inserted between the *Sfo*I and *Bsm*BI sites of pBS1 [3], replacing *LEU2* with *hphMX*, creating pMJ734. A PCR fragment carrying *TRP1* was ligated between the *Xma*I and *Ppu*MI sites of this plasmid, deleting the *CEN*-*ARS* region. The resulting plasmid (pMJ772) was integrated at *TRP1* by digesting with *Bsu*36I, transforming and selecting for hygromycin resistance. To create control strains carrying *SGS1* at *TRP1*, the *Xho*I-*Sac*I fragment from pSM100 [3] was inserted between the *Xho*I and *Sac*I sites of pMJ734, and *TRP1* was then inserted between *Xma*I and *Ppu*MI to create pMJ771. This plasmid was integrated at *TRP1* as described above. The meiotic null allele of *SGS1* (*sgs1-mn*) was made as described [4]. It replaces the normal *SGS1* promoter with the following, in 5’ to 3’ order: a *kanMX* G418^r^ cassette; the *CLB2* promoter; and an amino-terminal HA-tag to allow protein detection.

**Random spore analysis***.* Map distances determined by random spore analysis used spores isolated as described [5]; map distances and standard error attributable to sample size were calculated using the following formula:

$$\begin{matrix} X=100\times r/t \\ S.E.=100\times\sqrt{(r/t)(1-r/t)/t} \end{matrix}$$

### where X = map distance in cM, S.E. = standard error of map distance, *r* = number of recombinant colonies counted and *t* = total number of colonies counted.

### **Detection of Sgs1p on Western blots.** Protein extracts were prepared from 1 ml of a meiotic culture essentially as described [6]. Samples equivalent to 0.02-0.03 ml of culture were displayed on 4-12% polyacrylamide tris-glycine gradient gels (Novex), with electrophoresis as recommended by the manufacturer. Proteins were then electoblotted to PVDF filters (Invitrogen) as recommended by the manufacturer. Filters were probed with mouse monoclonal anti-HA (clone 12CA5; Roche), followed by goat anti-mouse antisera conjugated to alkaline phosphatase (Sigma), and the signal was developed using the chemiluminescent CPD-star substrate (Applied Biosystems). Chemiluminsescence was detected using a Fuji FLA1000 CCD camera, and the resulting images quantified using Fuji ImageGuage software.

### Supplementary Online References

1. Becker DM, Guarente L (1991) High-efficiency transformation of yeast by electroporation. Methods Enzymol 194: 182-187.

2. Goldstein AL, McCusker JH (1999) Three new dominant drug resistance cassettes for gene disruption in *Saccharomyces cerevisiae*. Yeast 15: 1541-1553.

3. Mullen JR, Kaliraman V, Brill SJ (2000) Bipartite structure of the *SGS1* DNA helicase in *Saccharomyces cerevisiae*. Genetics 154: 1101-1114.

4. Lee BH, Amon A (2003) Role of Polo-like kinase *CDC5* in programming meiosis I chromosome segregation. Science 300: 482-486.

5. Rockmill B, Lambie EJ, Roeder GS (1991) Spore enrichment. Methods Enzymol 194: 146-149.

6. Foiani M, Marini F, Gamba D, Lucchini G, Plevani P (1994) The B subunit of the DNA polymerase alpha-primase complex in *Saccharomyces cerevisiae* executes an essential function at the initial stage of DNA replication. Mol Cell Biol 14: 923-933.

7. Allers T, Lichten M (2001) Differential timing and control of noncrossover and crossover recombination during meiosis. Cell 106: 47-57.

8. Jessop L, Allers T, Lichten M (2005) Infrequent co-conversion of markers flanking a meiotic recombination initiation site in *Saccharomyces cerevisiae*. Genetics 169: 1353-1367.

9. Rockmill B, Roeder GS (1990) Meiosis in asynaptic yeast. Genetics 126: 563-574.
