# Supplementary material for: Meiotic Chromosome Synapsis-Promoting Proteins Antagonize the Anti-Crossover Activity of Sgs1
Source: PLoS Genet. 2006 Sep 22;2(9):e155. doi: 10.1371/journal.pgen.0020155 (PMC1570379; doi:10.1371/journal.pgen.0020155)
Supplement: Supplementary file 2 [file pgen.0020155.st001.doc]

**Table S1.** Strain Genotypes

| Name | Genotype |
| --- | --- |
|  |  |
| A. SK1 strains | |
|  | |
| 1. Derived from MJL2442 | |
|  |  |
| MJL2442 | *his4::URA3-arg4-EcPal(1691) LEU2 MATa* |
|  | HIS4 leu2::URA3-ARG4 MATα |
|  |  |
| MJL3035 | MJL2442 *sgs1∆::KanMX TRP1::hphMX::SGS1* |
|  | *sgs1∆::KanMX TRP1::hphMX::SGS1* |
|  |  |
| MJL3039 | MJL2442 *sgs1∆::KanMX TRP1::hphMX::sgs1∆C795* |
|  | *sgs1∆::KanMX TRP1::hphMX::sgs1∆C795* |
|  |  |
| MJL3065 | MJL2442 *sgs1∆::KanMX TRP1::hphMX::SGS1 zip1∆(EcoR1-Spe1)::lys2(XbaI-BglII)::KanMX6* |
|  | *sgs1∆::KanMX TRP1::hphMX::SGS1 zip1∆(EcoR1-Spe1)::lys2(XbaI-BglII)::KanMX6* |
|  |  |
| MJL3064 | MJL2442 *sgs1∆::KanMX TRP1::hphMX::sgs1∆C795 zip1∆(EcoR1-Spe1)::lys2(XbaI-BglII)::KanMX6* |
|  | *sgs1∆::KanMX TRP1::hphMX::sgs1∆C795 zip1∆(EcoR1-Spe1)::lys2(XbaI-BglII)::KanMX6* |
|  |  |
| MJL3069 | MJL2442 *sgs1∆::KanMX TRP1::hphMX::SGS1 zip2∆::natMX* |
|  | *sgs1∆::KanMX TRP1::hphMX::SGS1 zip2∆::natMX* |
|  |  |
| MJL3070 | MJL2442 *sgs1∆::KanMX TRP1::hphMX::sgs1∆C795 zip2∆::natMX* |
|  | *sgs1∆::KanMX TRP1::hphMX::sgs1∆C795 zip2∆::natMX* |
|  |  |
| MJL3073 | MJL2442 *sgs1∆::natMX TRP1::hphMX::SGS1 msh4∆::kanMX* |
|  | *sgs1∆::natMX TRP1::hphMX::SGS1 msh4∆::kanMX* |
|  |  |
| MJL3120 | MJL2442 *SGS1 TRP1 msh4∆::kanMX* |
|  | *SGS1 TRP1 msh4∆::kanMX* |
|  |  |
| MJL3074 | MJL2442 *sgs1∆::natMX TRP1::hphMX::sgs1∆C795 msh4∆::kanMX* |
|  | *sgs1∆::natMX TRP1::hphMX::sgs1∆C795 msh4∆::kanMX* |
|  |  |
| MJL3075 | MJL2442 *sgs1∆::KanMX TRP1::hphMX::SGS1 mer3∆::natMX* |
|  | *sgs1∆::KanMX TRP1::hphMX::SGS1 mer3∆::natMX* |
|  |  |
| MJL3076 | MJL2442 *sgs1∆::KanMX TRP1::hphMX::sgs1∆C795 mer3∆::natMX* |
|  | *sgs1∆::KanMX TRP1::hphMX::sgs1∆C795 mer3∆::natMX* |
|  |  |
| MJL3091 | MJL2442 *KanMX::pCLB2-3HA-SGS1* |
|  | *KanMX::pCLB2-3HA-SGS1* |
|  |  |
| MJL3124 | MJL2442 *KanMX::pCLB2-3HA-SGS1 msh4∆* |
|  | *KanMX::pCLB2-3HA-SGS1 msh4∆* |
|  |  |
| BR4593 | MJL2442 *sgs1∆::KanMX TRP1::hphMX::SGS1 ZIP3-GFP::URA3* |
|  | *sgs1∆::KanMX TRP1::hphMX::SGS1 ZIP3* |
|  |  |
| BR4594 | MJL2442 *sgs1∆::KanMX TRP1::hphMX::sgs1∆C795 ZIP3-GFP::URA3* |
|  | *sgs1∆::KanMX TRP1::hphMX::sgs1∆C795 ZIP3* |
|  |  |
| 2. Derived from MJL2984 | |
|  |  |
| MJL2984 | *his4::URA3-tel-arg4-EcPal+9 LEU2_______________* |
|  | *HIS4 leu2:URA3-tel-ARG4* |
|  |  |
| MJL3033 | MJL2984 *sgs1∆::KanMX TRP1::hphMX::SGS1* |
|  | *sgs1∆::KanMX TRP1::hphMX::SGS1* |
|  |  |
| MJL3034 | MJL2984 *sgs1∆::KanMX TRP1::hphMX::sgs1∆C795* |
|  | *sgs1∆::KanMX TRP1::hphMX::sgs1∆C795* |
|  |  |
|  |  |
|  |  |
| B. BR strains | |
|  |  |
| BR3485 | *HIS4 leu2-3,112 MAT***a** *ade2-1 thr1-4 trp1-289 ura3-1 lys2-99* |
|  | *his4-260,519 LEU2 MATα ade2-1 thr1-4 trp1-289 ura3-1 lys2-99* |
|  |  |
| BR3486 | BR3485 *sgs1∆::kanMX* |
|  | *sgs1∆::kanMX* |
|  |  |
| BR3616 | BR3485 *msh4∆::ADE2* |
|  | *msh4∆::ADE2* |
|  |  |
| BR3617 | BR3485 *msh4∆::ADE2* *sgs1∆::kanMX* |
|  | *msh4∆::ADE2* *sgs1∆::kanMX* |
|  |  |
| BR3643 | BR3485 *zip3∆::URA3* |
|  | *zip3∆::URA3* |
|  |  |
| BR3644 | BR3485 *zip3∆::URA3 sgs1∆::kanMX* |
|  | *zip3∆::URA3 sgs1∆::kanMX* |
|  |  |
| BR3677 | BR3485 *zip1::LYS2* |
|  | *zip1::LYS2* |
|  |  |
| BR3668 | BR3485 *zip1::LYS2 sgs1∆::kanMX* |
|  | *zip1::LYS2 sgs1∆::kanMX* |
|  |  |
|  |  |

Supplementary Table 1. SK1 strains are derived from MJL2442 [7] or MJL2984 (haploid parents described in [8]) with the indicated additional markers, and are also homozygous *ura3 arg4 lys2 ho::LYS2*. BR strains strains are derived from BR1919-8B [9].
