# Supplementary material for: Meiotic Chromosome Synapsis-Promoting Proteins Antagonize the Anti-Crossover Activity of Sgs1
Source: PLoS Genet. 2006 Sep 22;2(9):e155. doi: 10.1371/journal.pgen.0020155 (PMC1570379; doi:10.1371/journal.pgen.0020155)
Supplement: Supplementary file 3 [file pgen.0020155.st002.doc]

### **Table S2.** *sgs1∆* Restores Crossovers to a *zip1∆* Mutant in the BR Strain Background.

|  | Map distance (cM) | | Total |
| --- | --- | --- | --- |
|  |  |  |  |
| Genotype | *HIS4-LEU2* | *LEU2-MAT* | analyzed |
|  |  |  |  |
| wild-type | 20.8 ± 1.7 | 28.4 ± 1.9 | 591 |
|  |  |  |  |
| *sgs1∆* | 25.5 ± 2.7 | 33.2 ± 2.9 | 259 |
|  |  |  |  |
| *zip1∆* | 7.5 ± 2.1 | 21.7 ± 3.3 | 161 |
|  |  |  |  |
| *zip1∆ sgs1∆* | 17.5 ± 2.3 | 33.6 ± 2.8 | 280 |
|  |  |  |  |

Map distances and calculated standard errors were determined by random spore analysis (see Materials and Methods and Protocol S1).
